# The Effect of Selenium Supplementation on Glucose Homeostasis and the Expression of Genes Related to Glucose Metabolism

**DOI:** 10.3390/nu8120772

**Published:** 2016-12-13

**Authors:** Ewa Jablonska, Edyta Reszka, Jolanta Gromadzinska, Edyta Wieczorek, Magdalena B. Krol, Sara Raimondi, Katarzyna Socha, Maria H. Borawska, Wojciech Wasowicz

**Affiliations:** 1 Nofer Institute of Occupational Medicine, Department of Toxicology and Carcinogenesis, Sw. Teresy 8 Street, 91-348 Lodz, Poland; edyta.reszka@imp.lodz.pl (E.R.); edyta.wieczorek@imp.lodz.pl (E.W.); 2 Nofer Institute of Occupational Medicine, Department of Biological and Environmental Monitoring, Sw. Teresy 8 Street, 91-348 Lodz, Poland; jolanta.gromadzinska@imp.lodz.pl (J.G.); magdalena.krol@imp.lodz.pl (M.B.K.); wojciech.wasowicz@imp.lodz.pl (W.W.); 3 European Institute of Oncology, Division of Epidemiology and Biostatistics, via Ripamonti 435, Milan 20139, Italy; sara.raimondi@ieo.it; 4 The Medical University of Bialystok, Department of Bromatoloy, A. Mickiewicza 2D Street, 15-222 Bialystok, Poland; katarzyna.socha@umb.edu.pl (K.S.); borawska@umb.edu.pl (M.H.B.)

**Keywords:** selenium, gene expression, insulin signaling, glucose metabolism, glycolysis, pyruvate metabolism, energy metabolism, glycated hemoglobin, fasting plasma glucose

## Abstract

The aim of the study was to evaluate the effect of selenium supplementation on the expression of genes associated with glucose metabolism in humans, in order to explain the unclear relationship between selenium and the risk of diabetes. For gene expression analysis we used archival samples of cDNA from 76 non-diabetic subjects supplemented with selenium in the previous study. The supplementation period was six weeks and the daily dose of selenium was 200 µg (as selenium yeast). Blood for mRNA isolation was collected at four time points: before supplementation, after two and four weeks of supplementation, and after four weeks of washout. The analysis included 15 genes encoding selected proteins involved in insulin signaling and glucose metabolism. In addition, HbA1c and fasting plasma glucose were measured at three and four time points, respectively. Selenium supplementation was associated with a significantly decreased level of HbA1c but not fasting plasma glucose (FPG) and significant down-regulation of seven genes: *INSR*, *ADIPOR1*, *LDHA*, *PDHA*, *PDHB*, *MYC*, and *HIF1AN*. These results suggest that selenium may affect glycemic control at different levels of regulation, linked to insulin signaling, glycolysis, and pyruvate metabolism. Further research is needed to investigate mechanisms of such transcriptional regulation and its potential implication in direct metabolic effects.

## 1. Introduction

Selenium (Se) is a trace element with multiple biological functions. The majority of studies related to this nutrient and human health were devoted to its potential anticancer activity and chemoprevention based on Se supplementation [1,2,3]. However recent scientific focus has been switched towards possible harmful effects of Se supplementation, especially in terms of its unclear link with glucose metabolism and diabetes [4,5,6]. The increased risk of diabetes type II (T2DM) in humans associated with elevated Se status has been suggested for the first time on the basis of reanalysis of a placebo-controlled randomized trial (NPC, Nutritional Prevention of Cancer) conducted in the USA in the years 1983–1991 in a group of 1312 subjects. In that study, there was a significant increase in the risk of diabetes among Se-supplemented subjects who had the highest Se status (plasma concentration >121.6 ng/mL) before supplementation (Risk Ratio (RR) = 2.40; *p* = 0.01) [7]. In another American study (SELECT, Selenium and Vitamin E Cancer Prevention Trial) conducted in 35,533 individuals, there was a non-significant increase in the risk of diabetes among Se- supplemented arm (RR = 1.07%, *p* = 0.16) [8]. Results of this large and well-designed, randomized, placebo-controlled trial generated much controversy and up to now, no clear conclusion have been made with respect to the potentially diabetogenic properties of Se. Several observational studies, as well as Se supplementation trials, in which the association between Se status/Se intake and the risk of diabetes or markers of glucose metabolism has been investigated, generated inconsistent results [4,9,10,11,12]. On the other hand, animal studies indicated that Se supplementation may lead to hyperinsulinemia, insulin resistance, and glucose intolerance [13]. To complicate this, previous in vivo studies indicated protective role of Se in diabetes [14,15]. The link between Se and glucose seems very complex and is supposed to be reflected by a non-linear U-shaped dose-response relationship [16].

The insights from experimental studies in animals indicate that Se is involved in glucose metabolism via Se-dependent proteins (selenoproteins), which possess redox properties. It is assumed that they may influence the insulin-dependent metabolic pathways, because both insulin release and insulin signaling are regulated by the cellular redox potential [17]. In particular, the essential role in the regulation of both processes is attributed to hydrogen peroxide, which is one of the reactive oxygen species produced inter alia by dismutation of superoxide anion radical (catalyzed by superoxide dismutase, SOD). Hydrogen peroxide is reduced to water with the participation of enzymes, such as Se-dependent glutathione peroxidase (GPx) and catalase (CAT). The role of GPx in the metabolism of insulin appears to be significant and complex, which is reflected by a relatively low expression of mRNA for GPx1 and its low enzymatic activity in the pancreas (1%–5% of the value observed in the liver; the study in mice) [18]. Moreover, it was shown that global overexpression of GPx1 mRNA in mice fed with a normal Se diet (0.4 mg/kg) was associated with hyperglycemia, hyperinsulinemia, and an increase in plasma leptin concentrations [19]. Another selenoprotein potentially important in the etiology of diabetes is selenoprotein P (Sepp1) which is produced mainly in the liver and secreted into plasma, acting as a Se transporter, and having also antioxidant properties. Misu et al. observed a positive correlation between the level of Sepp1 mRNA and insulin resistance in humans [20]. The same authors also demonstrated in vitro that cells stimulated exogenously with purified Sepp1 were characterized by impaired insulin signaling and impaired glucose metabolism [20]. Recently it has been also observed that serum concentration of Sepp1 was positively correlated with fasting plasma glucose (FPG) concentration (*r* = 0.35, *p* = 0.037) and negatively correlated with serum adiponectin levels (*r* = −0.355, *p* = 0.034; *r* = −0.367, *p* = 0.028) in patients with T2DM [21].

Altogether, mechanisms of action of Se in humans at the level of glucose metabolism and its potential role in the etiology of diabetes are unknown and point to the need for more studies relevant to human metabolism. The aim of this study was to assess the effect of Se supplementation on the expression of genes related to glucose metabolism, including genes encoding hormones responsible for glycemic control (insulin, glucagon, leptin, and adiponectin), receptors for these hormones, enzymes involved in glycolysis, as well as key transcription factors involved in the regulation of glycolysis. To our knowledge this is the first study conducted in humans, which aimed to investigate more in-depth molecular effects of Se with respect to glucose homeostasis.

## 2. Materials and Methods 

### 2.1. Study Design

For the purpose of this study we used archival samples of cDNA obtained from subjects who were supplemented with Se during the trial conducted by us previously. Details of the trial, as well as subjects’ exclusion criteria, were described elsewhere [22]. The exclusion criteria for subjects’ recruitment included inter alia current smoking and self-reported prevalence of diabetes. Briefly, the 95 non-smoking and non-diabetic individuals were supplemented with 200 µg of Se/day (in the form of Se yeast) for six weeks and fasting blood was collected at four time points: at baseline, after two weeks of supplementation, after six weeks of supplementation, and after four weeks of washout. In the present study, gene expression analysis was performed with the use of cDNA obtained from 76 subjects, randomly selected out of the entire study group, including 36 males and 40 females. Table 1 presents basic characteristics of the subjects. Plasma Se concentration was measured previously (as described in [22]) and mean plasma Se levels calculated for 76 subjects at each time points are presented also in Table 1.

Written informed consent was obtained from all the study participants and the study was approved by the Local Ethics Committee (Ethical Institutional Review Board at the Nofer Institute of Occupational Medicine, Lodz, Poland, Resolution No. 3/2010).

### 2.2. Gene Expression Experiment

mRNA for gene expression analysis was isolated from white blood cells (WBC). The description of material preservation, mRNA isolation, quantification, and quality assessment were described previously [22]. For reverse transcription we used a QuantiTect Kit (Qiagen, Hilden, Germany). Gene expression was analyzed by means of qPCR and conducted on a QuantStudioTM 12K Flex Real-Time PCR System (Thermo Fisher Scienific, Waltham, MA, USA), using Custom Taqman^®^ array cards and TaqMan^®^ Universal Master Mix (Thermo Fisher Scienific, Waltham, MA, USA). For each array card 6 µg of cDNA was taken. Reaction conditions were as follows: 95 °C of initial denaturation for 10 min followed by 50 cycles of 95 °C for 15 s and 60 °C for 60 s. The analysis included 15 genes encoding selected proteins involved in insulin signaling and glucose metabolism. The expression was normalized against *GAPDH*. The list of all targets is presented in Table 2. The genes were divided into four groups according to the type of encoded protein: (1) hormones: insulin (*INS*), glucagon (*GCG*), adiponectin (*ADIPOQ*), and leptin (*LEP*); (2) hormone receptors: insulin receptor (*INSR*), glucagon receptor (*GCGR*), adiponectin receptors (*ADIPOR1*, *ADIPOR2*), and leptin receptor (*LEPR*); (3) enzymes involved in pyruvate metabolism: lactic acid dehydrogenase (*LDHA*), pyruvate dehydrogenase, alpha subunit (*PDHA1*) and beta subunit (*PDHB*); (4) transcription factors or their inhibitors driving glycolysis: transcription factor HIF1 (*HIF1A*), HIF1A inhibitor (*HIF1AN*), and nuclear transcription factor MYC (*MYC*).

### 2.3. HbA1c, Fasting Plasma Glucose

Levels of HbA1c (glycated hemoglobin) were determined in 10 µL of whole blood (heparinized tube) immediately after the blood was collected. The analysis was performed commercially in the certified laboratory of clinical diagnostics, using immunoturbidimetric assay on an INTEGRA 400 system (Roche Diagnostics, Rotkreuz, Switzerland). The enzymic colorimetric method was based on an oxidase/peroxidase method and the respective commercial kit (Alpha Diagnostics, Warsaw, Poland) was used to analyze FPG. All absorbance values were measured on a UV4 Unicam UV-VIS spectrophotometer (Cambridge, UK). Intra- and inter-assay coefficients of variation for FPG analyses were 2.52% (*n* = 10) and 4.57% (*n* = 32), respectively.

### 2.4. Statistical Analysis

The analysis on gene expression data was performed on the log-transformed values in order to have a normal distribution. Repeated measures analysis of variance (MANCOVA) with an unstructured covariance matrix was carried out to test the effects of time on FPG, HbA1c, and gene expression. With this analysis, markers (FPG, HbA1c, gene expression) at different time points were considered as dependent variables, while covariates (age, sex, BMI, and baseline Se) were included in the models as independent variables. The possible correlation between variation of Se and of gene expression values at different time points was investigated by a linear regression model with the difference of gene expression at two time points as the dependent variable, and the difference of Se, age, sex, BMI, baseline Se, and baseline gene expression as independent variables. All of the analyses were carried out in the whole group of subjects and separately in women and men. Post-hoc comparisons between pairwise time points were performed by contrast tests, using Bonferroni correction. All of the other statistical tests were performed at a significance level of α = 0.05. To adjust the results for multiple comparisons, we performed a false discovery rate (FDR) analysis. Statistical analyses were conducted using SAS software, version 9.2 (SAS Institute, Cary, NC, USA).

## 3. Results

### 3.1. The Effect of Se Supplementation on HbA1c and Fasting Plasma Glucose

Significant changes in HbA1c levels were observed upon Se supplementation (*p* < 0.0001), with post hoc tests indicating a significant decrease both after six weeks of supplementation and after four weeks of washout (*p* < 0.0001 and *p* = 0.02 vs. baseline, respectively) (Table 3). On the contrary, FPG concentration remained unaffected upon supplementation trial (*p* = 0.553).

### 3.2. The Effect on Gene Expression

In the first step of analysis, we excluded genes which were not expressed or which had very low expression. These were five genes: *INS*, *GCG*, *GCGR*, *ADIPOQ*, and *LEP*. Further analysis of the remaining 10 genes indicated a significant reduction in mRNA expression of seven targets, including *INSR*, *ADIPOR1*, *LDHA*, *PDHA*, *PDHB*, *MYC*, and *HIF1AN*. In the case of *INSR*, *LDHA*, *PDHA*, and *MYC*, a significant decrease was observed at all measurement points as indicated by contrast tests (during supplementation and the washout period). For genes *ADIPOR2*, *LEPR*, and *HIF1A* there was no statistically significant changes over time (Table 3).

Further analysis aimed to test whether the variation of gene expression depended on the variation of Se. We conducted this analysis for all 10 genes and all time points regardless of the fact whether the overall effect was significant or not. Significant correlations between Se status and gene expression were found in the case of *ADIPOR1* after six weeks of supplementation (*p* = 0.012) and in the case of *HIF1AN* after two weeks (*p* = 0.045) and six weeks (*p* = 0.002) of supplementation. All data are shown in Table S3.

### 3.3. Sex Differences

At baseline, males had significantly higher levels of FPG (*p* = 0.01) as compared to females, however, both sexes did not differ significantly in the level of HbA1c (*p* = 0.175). No modifying effect of sex was observed at the level of both markers upon time (during supplementation and after washout period, as assessed in the MANCOVA model; *p* = 0.55 and *p* = 0.09 for time interaction with sex for FPG and HbA1c, respectively). Data on FPG, HbA1c, and on gene expression analyzed separately for males and females are presented in the supplementary materials (Tables S1 and S2). The results of analysis stratified by sex were less powered and some *p*-values were no more significant. However, general trends were similar. Analogically to the analyses conducted in the whole group, we tested the correlation between variations in plasma Se levels and in gene expression. In males significant correlation was found for *HIF1AN* after six weeks of supplementation (*p* = 0.038). In females significant correlations were observed for four genes after six weeks of supplementation: *ADIPOR1* (*p* = 0.047), *PDHB* (*p* = 0.034), *HIF1AN* (*p* = 0.035), and *MYC* (*p* = 0.048). Regression analysis data are shown in Tables S4 (for males) and S5 (for females).

### 3.4. False Discovery Rate Analysis

*P*-values for the effect on gene expression (Table 3, Tables S1 and S2) were still statistically significant after FDR adjustment for multiple comparisons, except for two genes in women: *ADIPOR1* and *HIF1AN* (in both cases the FDR-adjusted *p*-value was 0.06).

## 4. Discussion

In this study we observed that six weeks of supplementation with 200 µg of Se in the form of Se yeast had significantly decreased the level of HbA1c in the subjects with relatively low baseline Se status. On the other hand we did not observe any changes at the level of FPG. However, FPG reflects the glycemic status at the time of measurement. HbA1c is regarded as a more reliable marker since it indicates long-term changes in the glycemic control. Thus, the impact of Se supplementation on HbA1c levels observed in this study confirms that Se is involved in the metabolism of glucose. Moreover, significant decrease in HbA1c levels was maintained four weeks after supplementation discontinuation, which clearly shows that the effect of Se on the glycemic control was relatively strong. There are only a few other studies reporting the effect of Se supplementation on glucose homeostasis in humans. Three of them, being all randomized, placebo-controlled trials (RCTs), have shown beneficial effect of short-term Se supplementation in specific groups of women. In the first study, 70 women with gestational diabetes were supplemented with Se at a dose of 200 µg per day for six weeks. Improved glucose homeostasis was observed as reflected by decreased levels of FPG and serum insulin as well as decreased HOMA-IR values (homeostasis model of assessment of insulin resistance) [23]. The second study was conducted in 70 women with polycystic ovary syndrome. After eight weeks of supplementation with Se at 200 µg per day, there was a significant decrease in serum insulin levels, HOMA-IR, HOMA-B (homeostatic model of assessment of beta-cell function) values, and an increase in QUICKI (quantitative insulin sensitivity check index), whereas FPG remained unchanged [24]. The last study investigated the effect of Se supplementation in women with central obesity. After six weeks of Se supplementation (200 µg/day), significant decrease in serum insulin levels and HOMA-IR values was observed [25]. Another study which was conducted in 60 subjects of both sexes (20 males and 40 females) diagnosed with T2DM and coronary heart disease also showed beneficial effects of Se supplementation (200 µg/day for eight weeks) in terms of decreased insulin, HOMA-IR, HOMA-B parameters, and increased QUICKI [26]. On the contrary, only one RCT, conducted in patients with T2DM (*n* = 60, including 34 males and 26 females), indicated that three months of Se supplementation (200 µg/day) led to the increased FPG levels [27]. To compare with short term supplementation, long-term RCTs did not indicate any effects on glucose homeostasis upon Se supplementation, as shown at the level of serum glucose concentration in 140 men with prostate cancer (supplemented with Se at dose 200 µg/day for five years) or at the level of FPG in 3146 participants of the SU.VI.MAX trial (supplemented with 100 µg of Se in combination with zinc (Zn) and vitamins C, E, and beta-carotene, for 7.5 years) [28,29]. Overall, the inconsistent results of the above RCTs coincide with a lack of compliance in the observational studies investigating the link between Se and diabetes. These studies also suggest that Se-induced effects may largely depend on specific health conditions (notably none of them has been conducted in the general population). A similar conclusion has been drawn from recent studies in mice, which were fed with a high-fat diet in order to induce insulin resistance [30]. It was observed that pre-treatment with Se protected animals against developing insulin resistance, whereas post-treatment with Se exacerbated this metabolic disorder. The effects of pre-treatment and post-treatment with Se were different in the context of adipogenesis and lipolysis, showing increased adipocyte differentiation and fat accumulation in adipose tissue in pre-treated mice, whereas post-treated mice were characterized by increased lipolysis and ectopic lipid deposition [30]. The above study clearly shows that Se exerts differential effects under different health conditions.

Along with HbA1c decrease upon Se supplementation, we have observed in this study a significant decrease of leukocyte mRNA levels for seven genes involved in different steps of glucose metabolism regulation. The first group of mRNA targets (*INSR* and *ADIPOR1*) concerned receptors for endocrine hormones: insulin and adiponectin, which are key players in insulin signaling responsible for cellular glucose uptake. Suppressive effects of Se on insulin receptors was observed previously in a study of gestating rats and their offspring [31]. In that experiment, decreased mRNA expression for *Insr*, as well as other insulin signaling genes, including *Irs-1* (insulin receptor substrate 1), *Irs-2* (insulin receptor substrate 2), and *Akt2* (serine/threonine protean kinase 2), was observed in the liver of dams supplemented with 3 mg Se/kg diet (as compared to dams fed basal diet). Importantly, similar changes in the liver, additionally accompanied by decreased protein levels of insulin receptors, were observed in the offspring of dams also receiving a high Se diet. At the same time, high Se diet was associated in both dams and the offspring with hyperinsulinemia, insulin resistance and glucose intolerance [31]. The impact of a high Se diet on protein levels for insulin receptors was also investigated in pigs, showing tissue-specific changes, with up-regulation in the liver and down-regulation in the muscle. However, no changes in both tissues were observed for insulin receptors at the level of mRNA [32]. Similarly, another study conducted in pigs also failed to show any effect of supranutritional intake of Se on insulin receptor mRNA levels, analyzed in liver, muscle, and visceral adipose tissue [33]. Significant reduction of mRNA expression for the adiponectin receptor, *ADIPOR1*, observed in our study after two weeks of supplementation, was accompanied by a borderline significant decrease in *ADIPOR2* (*p* = 0.08). AdipoR2 is expressed mainly in the liver; thus, in blood leukocytes it was more probable to observe a significant effect in the case of AdipoR1, which is expressed ubiquitously [34]. Both receptors bind adiponectin, a hormone produced by adipocytes and involved in glucose metabolism via modulating insulin sensitivity [34]. Though our study may suggest some effect of Se on adiponectin signaling, two supplementation trials failed to show any effect of Se administration on plasma adiponectin levels in humans [35,36]. Nevertheless, the suppressive effect on *INSR* and *ADIPOR1* indicated in this study deserves further research, as decreased expression of mRNA levels for these receptors has been linked to insulin resistance and diabetes in humans and animals [37,38,39]. Decreased expression of essential genes involved in glucose metabolism, such as those encoding receptors for insulin and adiponectin, may indicate rather detrimental effects of Se supplementation in the study group. On the other hand, the effect at the level of HbA1c was somewhat positive. The hypothetical explanation for these contradictory observations may be that the potentially beneficial decrease in HbA1c level reflects an adaptive response which counteracts the adverse effects of the metalloid on glucose homeostasis and that this response is rather short-term. This adaptive mechanism when induced in a constant manner (by introducing the factor day by day for several weeks) may be strong enough to influence the level of HbA1c, however, not in the long-term. The hypothesis about short-term adaptive response is in line with overall observations from Se supplementation trials, indicating mainly beneficial effects of Se on glucose metabolism in the case of short-term administration and, on the other hand, null or negative effects upon long-term supplementation with this nutrient. Alternative explanation for decreased levels of HbA1c upon Se is the possibility that Se may prevent the process of hemoglobin glycation. This may be suggested on the basis of a recent study by Yu and colleagues, showing Se nanoparticles prevent protein glycation in vitro [40]. Nevertheless, it is not clear whether changes observed in our study at the level of leukocyte gene expression reflect changes in insulin-sensitive tissues. If they do, down-regulation of *INSR* and *ADIPOR1* in blood leukocytes could serve as an early biomarker of adverse health effects of Se supranutrition in humans, leading to impaired glucose homeostasis.

The second group of down-regulated genes in this study included *LDH*, *PDHA*, and *PDHB*, all three encoding enzymes involved in pyruvate metabolism. Pyruvate is the end product of glycolysis and its further metabolism depends on cellular oxygen concentration. Under anaerobic conditions, it is transformed by lactate dehydrogenase (LDH) to lactic acid whereas, under normal aerobic conditions, it is metabolized by pyruvate dehydrogenase complex (PDC) to acetyl coenzyme A (acetyl CoA). The latter reaction links glycolysis to the Krebs cycle. Importantly, pyruvate recycling (occurring across mitochondrial membranes) is an important regulator of insulin secretion due to the generation of NADPH (the reduced form of nicotinamide adenine dinucleotide phosphate), the pivotal molecule required for insulin granule exocytosis in beta cells [41]. Pyruvate metabolism was shown to be implicated in diabetes, as reflected by increased activity of pyruvate dehydrogenase kinase (PDK) in diabetic subjects (PDK plays significant role in glucose disposal as it decreases the activity of PDC) [42]. In general, glycolytic pathway was shown to be dysregulated in diabetes [43]. A study in rats showed, for example, that an alloxan-induced diabetic state significantly altered the activities of glycolytic enzymes in lymphocytes [44]. Importantly, recent animal studies suggest that Se interferes with glycolytic targets and pyruvate metabolism, supporting our observation. Pigs fed with a supranutritional dose of Se exhibited decreased expression of mRNA for pyruvate kinase (enzyme responsible for pyruvate synthesis in the last step of glycolysis) [33]. Interestingly, increased pyruvate levels in the liver and increased expression of pyruvate metabolizing enzymes (pyruvate carboxylase (Pcx) and pyruvate dehydrogenase (Pdh)) were shown in mice with a knocked out gene for selenocysteine lyase (SCLY, the enzyme catalyzing conversion of selenocysteine into alanine and selenide, an important step in Se metabolism, as selenide is a substrate for selenoprotein biosynthesis) [45]. Recently it has been also shown that Se affects glycolysis in cancer cells, suggesting that down-regulation of glycolytic enzymes is a novel mechanism of Se toxicity in cancer and that this mechanism targets the well-known metabolic hallmark of cancer cells associated with enhanced glycolysis, which is called the Warburg effect [46]. In our study we have observed, additionally that Se may affect also transcriptional regulation of glycolysis, leading to significantly decreased expression of *MYC*. c-MYC protein, encoded by *MYC* gene, is responsible for transcriptional regulation of all glycolytic targets, including pyruvate kinase, pyruvate kinase dehydrogenase and lactate dehydrogenase [47]. Importantly, it was also shown to regulate the expression of glucose transporter 2 (GLUT2) and 4 (GLUT4) [47]. mRNA expression of the second major transcriptional regulator of glycolytic targets, HIF1 [47] was not affected upon Se supplementation in our study, whereas a significant decrease was observed for its inhibitor (but only after two weeks of supplementation). Altogether, these results suggest the need for a more in-depth investigation of the link between Se and glycolysis in humans.

We were not able to detect the expression of genes encoding: insulin (*INS*), glucagon (*GCG*), adiponectin (*ADIPOQ*), and leptin (*LEP*) in leukocytes. This was not surprising as all of these hormones are produced either by pancreas (insulin, glucagon) or by adipocytes (adiponectin, leptin). Similarly, *GCGR* levels were not detectable in leukocytes, which was also expected, as the glucagon receptor is expressed mainly in the liver, in which it mediates glucagon effects: glycogen breakdown and glucose release into the bloodstream.

The last analysis in this study concerned the correlation between the variation in gene expression and variation in plasma Se concentrations at a particular time point. There were only a few significant correlations and those that were significant were very small, indicating that changes in gene expression were not high enough to correlate with changes in Se.

Since this report presents secondary outcomes of the supplementation trial, it has several weaknesses. First of all, due to the short-term supplementation (six weeks) we were not able to assess changes in HbA1c levels after the whole life span of erythrocytes (which is about 120 days). HbA1c levels reflect the cumulative history of glycemic control in the past two to three months [48]. After this period the measurement of HbA1c levels is most indicative. However it is possible to observe changes earlier, as shown by studies investigating the effects of glucose-lowering drugs [49]. Since erythrocytes are produced constantly (meaning that the whole pool of erythrocytes contains cells of different ages) the effect observed in our study resulted from HbA1c reduction in the youngest erythrocytes. Nevertheless, one could speculate that these changes might have occurred due to some potential confounders present in the study during several weeks before the beginning of the trial, such as pharmacological or dietary factors. However, none of the supplemented subjects declared to be taking any drugs or supplements that could affect glucose metabolism (we asked all subjects about their use of glucose-lowering drugs or supplements before and during the trial). As for the influence of diet, none of the individuals reported a change to his/her dietary habits during the trial. The second major weakness of this study is attributable to the fact that the trial was not randomized (subjects were selected for the study according to genotype) and not placebo controlled (for details see [22]). Since no separate control group was investigated, we compared all data with respect to baseline values, measured on the first day of the trial, before taking the first pill with the supplement. Finally, gene expression was analyzed in this study in blood leukocytes and it is not clear whether these changes reflect general reactions to Se at the molecular level in humans or if they are tissue specific. In addition we did not analyze the protein levels for genes of interest; thus, we were unable to show any correspondence between transcriptomic and proteomic expression and elucidate whether observed subtle changes in mRNA have any further consequences.

## 5. Conclusions 

To conclude, results of this study suggest that Se may affect glycemic control at different levels of regulation, linked not only to insulin signaling, but also to glycolytic pathway and pyruvate metabolism. Further research is needed to investigate mechanisms of negative transcriptional regulation in blood leukocytes upon Se treatment, its relevance to other tissues, correspondence to protein levels and, finally, potential implications in direct metabolic and cellular effects. Overall, findings of this study added more insight into the controversial topic of possible diabetogenic effects of Se supplementation in humans. What is the exact impact of Se supplementation on glucose homeostasis and the risk of diabetes remains still an open question. The answer will not be obtained without studies unraveling the underlying mechanisms, as well as factors which may modify Se effects with respect to glucose homeostasis, in addition to these already suggested, such as sex and genotype [50,51], as well as Se speciation [52,53]. Explaining the link between Se, glucose metabolism, and diabetes is currently one of the priority goals in Se research because it will determine the further direction of studies focusing on Se as a potential chemopreventive agent.

## Figures and Tables

**Table 1:** Basic characteristics of the study group (76 subjects supplemented with selenium).

Variable	All (*n* = 76)	Males (*n* = 36)	Females (*n* = 40)	*p* (Males vs. Females)
**Age (years)**	34.8 ± 10.4	35 ± 18	36 ± 18	0.7549 ^a^
**BMI (kg/m^2^)**	24.0 ± 2.9	25.0 ± 2.7	23.0 ± 2.9	**0.0009** ^a^
**Smoking, *n* (%)**				
Current	0 (0)	0 (0)	0 (0)	
Ever	18 (24)	8 (23)	10 (24)	
Never	58 (76)	27 (77)	31 (76)	ns
**Use of Se-containing supplements in the past 6 months, *n* (%)**				
Yes	5 (7)	3 (9)	2 (5)	
No	71 (93)	32 (91)	39 (95)	ns
**Mean plasma Se ± SD (µg/L)**				
Baseline	65.2 ± 16.5	65.2 ± 16.6	65.3 ± 16.5	0.9729 ^b^
After two weeks	101.1 ± 24.5	98.7 ± 22.8	103.3 ± 26.1	0.3907 ^a^
After six weeks	99.1 ± 20.3	95.0 ± 16.9	102.7 ± 22.4	0.0928 ^b^
After four weeks of washout	76.5 ± 15.6	75.3 ± 15.3	77.6 ± 16.1	0.7777 ^b^
***p* (ANOVA)**	**<0.0001**	**<0.0001**	**<0.0001**	

^a^—Mann-Whitney U test; ^b^—Student’s *t*-test. Significant *p* values are in bold.

**Table 2:** Genes of interest selected for the study.

Gene	Locus	Gene Product	Protein Group
***INS***	11p15.5	insulin	hormones
***GCG***	2q36-q37	glucagon	
***ADIPOQ***	3q27	adiponectin	
***LEP***	1p31	leptin	
***INSR***	19p13.3-p13.2	insulin receptor	hormone receptors
***GCGR***	17q25	glucagon receptor	
***ADIPOR1***	1q32.1	adiponectin receptor 1	
***ADIPOR2***	12p13.31	adiponectin receptor 2	
***LEPR***	7q31.3	leptin receptor	
***LDHA***	11p15.4	lactate dehydrogenase	enzymes involved in pyruvate metabolism
***PDHA1***	Xp22.1	pyruvate dehydrogenase (lipoamide) alpha 1 subunit	
***PDHB***	3p21.1-p14.2	pyruvate dehydrogenase (lipoamide) beta subunit	
***HIF1A***	14q23.2	hypoxia inducible factor 1, alpha subunit	transcription factors and their inhibitors, involved in the regulation of glycolysis
***HIF1AN***	10q24	hypoxia inducible factor 1, alpha subunit inhibitor	
***MYC***	8q24.21	v-myc avian myelocytomatosis viral oncogene homolog	

**Table 3:** The effect of Se supplementation on fasting plasma glucose concentration, HbA1c levels and gene expression. Data for all study subjects (*n* = 76).

Marker	Time Points				*p* (MANCOVA *)
	Baseline	Two Weeks	Six Weeks	Washout	
**FPG (mg/dL)**	91.96 ± 13.94	90.55 ± 14.07	91.00 ± 14.15	91.56 ± 13.79	0.55
**HbA1c (%)**	4.74 ± 0.80	na	**4.44 ± 0.63 ^c^**	**4.57± 0.65 ^a^**	**<0.05**
***INSR***	1.23 ± 0.67	**0.98 ± 0.25 ^b^**	**1.02 ± 0.27 ^b^**	**0.95 ± 0.25 ^c^**	**0.005**
***ADIPOR1***	2.05 ± 0.23	**2.00 ± 0.18 ^a^**	2.07 ± 0.21	2.04 ± 0.23	**0.002**
***ADIPOR2***	1.88 ± 0.23	1.84 ± 0.18	1.84 ± 0.19	1.80 ± 0.19	0.08
***LEPR***	0.87 ± 0.35	0.80 ± 0.34	0.80 ± 0.29	0.82 ± 0.38	0.26
***LDHA***	2.74 ± 0.30	**2.59 ± 0.26 ^c^**	**2.49 ± 0.21 ^c^**	**2.51 ± 0.27 ^c^**	**<0.0001**
***PDHA***	2.03 ± 0.29	**1.93 ± 0.24 ^a^**	**1.90 ± 0.20 ^c^**	**1.90 ± 0.26 ^b^**	**0.0002**
***PDHB***	1.92 ± 0.25	1.86 ± 0.20	**1.81 ± 0.18 ^c^**	**1.81 ± 0.21 ^b^**	**0.0008**
***HIF1A***	2.37 ± 0.26	2.29 ± 0.23	2.29 ± 0.24	2.30 ± 0.27	0.16
***HIF1AN***	1.71 ± 0.32	**1.61 ± 0.19 ^b^**	1.70 ± 0.20	1.67 ± 0.21	**0.0001**
***MYC***	2.31 ± 0.29	**2.22 ± 0.26 ^a^**	**2.14 ± 0.22 ^c^**	**2.18 ± 0.25 ^a^**	**<0.0001**

Values significantly different as compared to baseline are in bold, ^a^—*p* < 0.05, ^b^—*p* < 0.01, ^c^—*p* < 0.001; *—Multivariate analysis of covariance, model included age, sex, BMI and baseline selenium; na—not analyzed (HbA1c was not analyzed after two weeks of supplementation).

## Supplementary Materials

The following are available online at http://www.mdpi.com/2072-6643/8/12/772/s1, Table S1: The effect of Se supplementation on fasting plasma glucose concentration, HbA1c levels and gene expression. Data for male subjects (*n* = 36); Table S2: The effect of Se supplementation on fasting plasma glucose concentration, HbA1c levels and gene expression. Data for female subjects (*n* = 40); Table S3: Correlation between changes in Se and changes in gene expression measured between two different time points. Data for all subjects (*n* = 76); Table S4: Correlation between changes in Se and changes in gene expression measured between two different time points in male subjects (calculated only for genes which were shown to be significantly changed upon Se supplementation). Data for male subjects (*n* = 36); Table S5: Correlation between changes in Se and changes in gene expression measured between two different time points in all subjects (calculated only for genes which were shown to be significantly changed upon Se supplementation). Data for female subjects (*n* = 40).
