# Supplementary material for: The Effect of Selenium Supplementation on Glucose Homeostasis and the Expression of Genes Related to Glucose Metabolism
Source: Nutrients. 2016 Dec 13;8(12):772. doi: 10.3390/nu8120772 (PMC5188427; doi:10.3390/nu8120772)
Supplement: Supplementary file 1 [file nutrients-08-00772-s001.docx]

**Supplementary Materials: The Effect of Selenium Supplementation on Glucose Homeostasis and the Expression of Genes Related to Glucose Metabolism**

**Ewa Jablonska, Edyta Reszka, Jolanta Gromadzinska, Edyta Wieczorek, Magdalena B. Krol,
Sara Raimondi, Katarzyna Socha, Maria H. Borawska and Wojciech Wasowicz**

**Table S1.** The effect of Se supplementation on fasting plasma glucose concentration, HbA1c levels and gene expression. Data for male subjects (*n* = 36).

| **Marker** | **Time points** | | | | ***p* (MANCOVA *)** |
| --- | --- | --- | --- | --- | --- |
|  | **Baseline** | **Two weeks** | **Six weeks** | **Washout** |  |
| **FPG (mg/dL)** | 94.70 ± 9.12 | 93.10 ± 10.21 | 93.85 ± 9.23 | 94.90 ± 10.40 | 0.68 |
| **HbA1c (%)** | 4.73 ± 0.37 | na | 4.40 ± 0.33 | 4.57 ± 0.38 | 0.94 |
| ***INSR*** | 1.22 ± 0.66 | 0.96 ± 0.33 | 0.98 ± 0.32 | 0.90 ± 0.32 | 0.06 |
| ***ADIPOR1*** | 1.97 ± 0.33 | 1.95 ± 0.33 | 2.02 ± 0.35 | 1.98 ± 0.37 | 0.07 |
| ***ADIPOR2*** | 1.87 ± 0.35 | 1.85 ± 0.35 | 1.86 ± 0.34 | **1.77 ± 0.35 ^a^** | **0.03** |
| ***LEPR*** | 0.82 ± 0.31 | 0.78 ± 0.38 | 0.79 ± 0.28 | 0.77 ± 0.37 | 0.83 |
| ***LDHA*** | 2.70 ± 0.50 | 2.62 ± 0.51 | **2.51 ± 0.45 ^c^** | **2.47 ± 0.45 ^c^** | **<0.0001** |
| ***PDHA*** | 2.01 ± 0.39 | **1.93 ± 0.40 ^a^** | **1.91 ± 0.36 ^b^** | **1.85 ± 0.37 ^c^** | **0.007** |
| ***PDHB*** | 1.91 ± 0.36 | 1.86 ± 0.37 | **1.84 ± 0.34 ^a^** | **1.79 ± 0.35 ^c^** | **0.03** |
| ***HIF1A*** | 2.30 ± 0.45 | 2.29 ± 0.43 | 2.27 ± 0.43 | 2.25 ± 0.44 | 0.71 |
| ***HIF1AN*** | 1.69 ± 0.39 | **1.60 ± 0.31 ^a^** | 1.72 ± 0.33 | 1.67 ± 0.33 | **0.01** |
| ***MYC*** | 2.28 ± 0.43 | 2.20 ± 0.43 | **2.13 ± 0.41 ^c^** | **2.11 ± 0.42 ^c^** | **0.0003** |

Values significantly different as compared to baseline are in bold; ^a^—*p* < 0.05, ^b^—*p* < 0.01, ^c^—*p* < 0.001; *—Multivariate analysis of covariance, model included age, sex, BMI, and baseline selenium; na—not analyzed (HbA1c was not analyzed after two weeks of supplementation).

**Table S2.** The effect of Se supplementation on fasting plasma glucose concentration, HbA1c levels and gene expression. Data for female subjects (*n* = 40).

| **Gene** | **Time points** | | | | ***p* (MANCOVA *)** |
| --- | --- | --- | --- | --- | --- |
|  | **Baseline** | **Two weeks** | **Six weeks** | **Washout** |  |
| **FPG (mg/dL)** | 89.50 ± 9.02 | 88.26 ± 8.59 | 88.43 ± 9.99 | 88.56 ± 6.92 | 0.33 |
| **HbA1c (%)** | 4.62 ± 0.36 | na | **4.48 ± 0.41** | **4.57 ± 0.42** | **0.03** |
| ***INSR*** | 1.20 ± 0.62 | **1.00 ± 0.26 ^a^** | 1.05 ± 0.29 | **1.00 ± 0.25 ^a^** | **0.03** |
| ***ADIPOR1*** | 2.07 ± 0.38 | **2.01 ± 0.36 ^a^** | 2.07 ± 0.39 | 2.06 ± 0.40 | **0.04** |
| ***ADIPOR2*** | 1.86 ± 0.36 | 1.83 ± 0.33 | 1.82 ± 0.35 | 1.81 ± 0.33 | 0.61 |
| ***LEPR*** | 0.88 ± 0.38 | 0.81 ± 0.33 | 0.82 ± 0.36 | 0.82 ± 0.34 | 0.36 |
| ***LDHA*** | 2.70 ± 0.49 | **2.56 ± 0.46 ^b^** | **2.48 ± 0.45 ^c^** | **2.52 ± 0.47 ^b^** | **0.0002** |
| ***PDHA*** | 1.99 ± 0.41 | 1.94 ± 0.38 | **1.88 ± 0.37 ^b^** | 1.94 ± 0.40 | **0.01** |
| ***PDHB*** | 1.89 ± 0.39 | 1.85 ± 0.34 | **1.79 ± 0.33 ^c^** | 1.83 ± 0.34 | **0.01** |
| ***HIF1A*** | 2.39 ± 0.43 | 2.30 ± 0.43 | 2.31 ± 0.43 | 2.34 ± 0.44 | 0.17 |
| ***HIF1AN*** | 1.70 ± 0.37 | **1.62 ± 0.33 ^a^** | 1.67 ± 0.34 | 1.66 ± 0.35 | **0.04** |
| ***MYC*** | 2.31 ± 0.46 | 2.23 ± 0.43 | **2.15 ± 0.39 ^c^** | 2.25 ± 0.41 | **0.001** |

Values significantly different as compared to baseline are in bold, ^a^—*p* < 0.05, ^b^—*p* < 0.01, ^c^—*p* < 0.001; *—Multivariate analysis of covariance, model included age, sex, BMI, and baseline selenium; na—not analyzed (HbA1c was not analyzed after two weeks of supplementation).

**Table S3.** Correlation between changes in Se and changes in gene expression measured between two different time points. Data for all subjects (*n* = 76).

| **Gene** | **Change between particular time points** | | |
| --- | --- | --- | --- |
|  | **Two weeks and baseline** | **Six weeks and baseline** | **Washout and baseline** |
| ***INSR **** | ß = −0.00145, *p* = 0.536 | ß = −0.00099678, *p* = 0.494 | ß = −0.00015073, *p* = 0.933 |
| ***ADIPOR1 **** | ß = −0.00145, *p* = 0.321 | ß = −0.00283, ***p* = 0.012** | ß = −0.00178, *p* = 0.244 |
| ***ADIPOR2*** | ß = 0.00187, *p* = 0.393 | ß = −0.00237, *p* = 0.100 | ß = −0.00251, *p* = 0.180 |
| ***LEPR*** | ß = 0.00204, *p* = 0.400 | ß = 0.00019544, *p* = 0.895 | ß = 0.00102, *p* = 0.648 |
| ***LDHA **** | ß = 0.00249, *p* = 0.284 | ß = −0.00002178, *p* = 0.985 | ß = −0.00031077, *p* = 0.879 |
| ***PDHA **** | ß = 0.00061083, *p* = 0.776 | ß = −0.00139, *p* = 0.190 | ß = 0.0001300, *p* = 0.948 |
| ***PDHB **** | ß = 0.0000092, *p* = 0.996 | ß = −0.00153, *p* = 0.080 | ß = −0.00154, *p* = 0.319 |
| ***HIF1A*** | ß = 0.00001425, *p* = 0.994 | ß = −0.00170, *p* = 0.172 | ß = −0.00071950, *p* = 0.698 |
| ***HIF1AN **** | ß = −0.00294, ***p* = 0.045** | ß = −0.00320, ***p* = 0.002** | ß = −0.00142, *p* = 0.333 |
| ***MYC **** | ß = 0.00195, *p* = 0.370 | ß = −0.00181, *p* = 0.083 | ß = 0.00061677, *p* = 0.733 |

Significant *p*-values are in bold; *—These genes were shown to be significantly changed upon Se supplementation.

**Table S4.** Correlation between changes in Se and changes in gene expression measured between two different time points in male subjects (calculated only for genes which were shown to be significantly changed upon Se supplementation). Data for male subjects (*n* = 36).

| **Gene** | **Change between particular time points** | | |
| --- | --- | --- | --- |
|  | **Two weeks and baseline** | **Six weeks and baseline** | **Washout and baseline** |
| ***INSR*** | ß = −0.00132, *p* = 0.772 | ß = −0.00114, *p* = 0.680 | ß = −0.00191, *p* = 0.542 |
| ***ADIPOR1*** | ß = 0.00007281, *p* = 0.974 | ß = −0.00200, *p* = 0.284 | ß = −0.00033170, *p* = 0.881 |
| ***ADIPOR2 **** | ß = 0.0057, *p* = 0.081 | ß = −0.00304, *p* = 0.252 | ß = −0.00161, *p* = 0.570 |
| ***LEPR*** | ß = 0.00855, ***p* = 0.021** | ß = −0.00174, *p* = 0.445 | ß = −0.00124, *p* = 0.736 |
| ***LDHA **** | ß = 0.00537, *p* = 0.195 | ß = −0.00036401, *p* = 0.829 | ß = 0.00022000, *p* = 0.928 |
| ***PDHA **** | ß = 0.00083922, *p* = 0.820 | ß = −0.00064144, *p* = 0.693 | ß = −0.00023848, *p* = 0.923 |
| ***PDHB **** | ß = 0.00042529, *p* = 0.888 | ß = −0.00004268, *p* = 0.977 | ß = −0.00072108, *p* = 0.737 |
| ***HIF1A*** | ß = 0.00212, *p* = 0.495 | ß = −0.00045045, *p* = 0.812 | ß = −0.00056424, *p* = 0.814 |
| ***HIF1AN **** | ß = −0.00230, *p* = 0.207 | ß = −0.00367, ***p* = 0.038** | ß = −0.00007654, *p* = 0.971 |
| ***MYC **** | ß = 0.00187, *p* = 0.582 | ß = −0.00111, *p* = 0.544 | ß = 0.00074800, *p* = 0.762 |

Significant *p*-values are in bold; *—These genes were shown to be significantly changed upon Se supplementation.

**Table S5.** Correlation between changes in Se and changes in gene expression measured between two different time points in all subjects (calculated only for genes which were shown to be significantly changed upon Se supplementation). Data for female subjects (*n* = 40).

| **Gene** | **Change between particular time points** | | |
| --- | --- | --- | --- |
|  | **Two weeks and baseline** | **Six weeks and baseline** | **Washout and baseline** |
| ***INSR **** | ß = −0.00137, *p* = 0.541 | ß = −0.00092861,*p* = 0.597 | ß = −0.00000379, *p* = 0.999 |
| ***ADIPOR1 **** | ß = −0.00221, *p* = 0.541 | ß = −0.00289, ***p* = 0.047** | ß = −0.00350, *p* = 0.118 |
| ***ADIPOR2*** | ß = −0.00002059, *p* = 0.994 | ß = −0.00209, *p* = 0.233 | ß = −0.00364, *p* = 0.162 |
| ***LEPR*** | ß = −0.00222, *p* = 0.472 | ß = 0.00103, *p* = 0.609 | ß = 0.00197, *p* = 0.501 |
| ***LDHA **** | ß = 0.00088311, *p* = 0.741 | ß = −0.000088, *p* = 0.967 | ß = −0.00181, *p* = 0.614 |
| ***PDHA **** | ß = 0.00067950, *p* = 0.799 | ß = −0.00185, *p* = 0.230 | ß = 0.00006802, *p* = 0.984 |
| ***PDHB **** | ß = 0.00008955, *p* = 0.967 | ß = −0.00238, ***p* = 0.034** | ß = −0.00265, *p* = 0.264 |
| ***HIF1A*** | ß = −0.00117, *p* = 0.673 | ß = −0.00196, *p* = 0.237 | ß = −0.00095784, *p* = 0.752 |
| ***HIF1AN **** | ß = −0.00326, *p* = 0.167 | ß = −0.00285, ***p* = 0.035** | ß = −0.00240, *p* = 0.292 |
| ***MYC **** | ß = 0.00232, *p* = 0.442 | ß = −0.00261, ***p* = 0.048** | ß = −0.00045776, *p* = 0.866 |

Significant *p*-values are in bold; *—These genes were shown to be significantly changed upon Se supplementation.
